# Supplementary figures and images for: Insect diversity estimation in polarimetric lidar
Source: PLoS One. 2024 Nov 1;19(11):e0312770. doi: 10.1371/journal.pone.0312770 (PMC11530007; doi:10.1371/journal.pone.0312770)

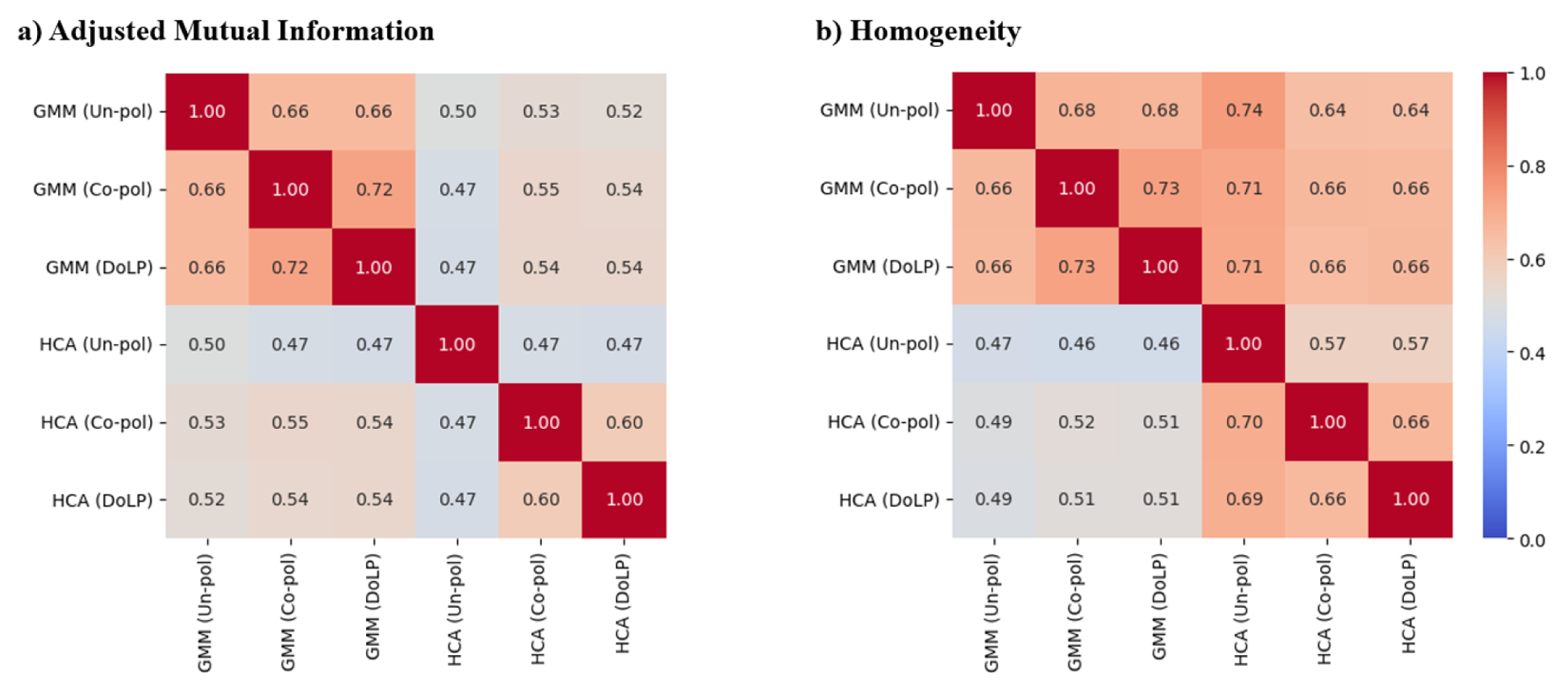

Supplement: S1 Fig — (TIF) [file pone.0312770.s001.tif]

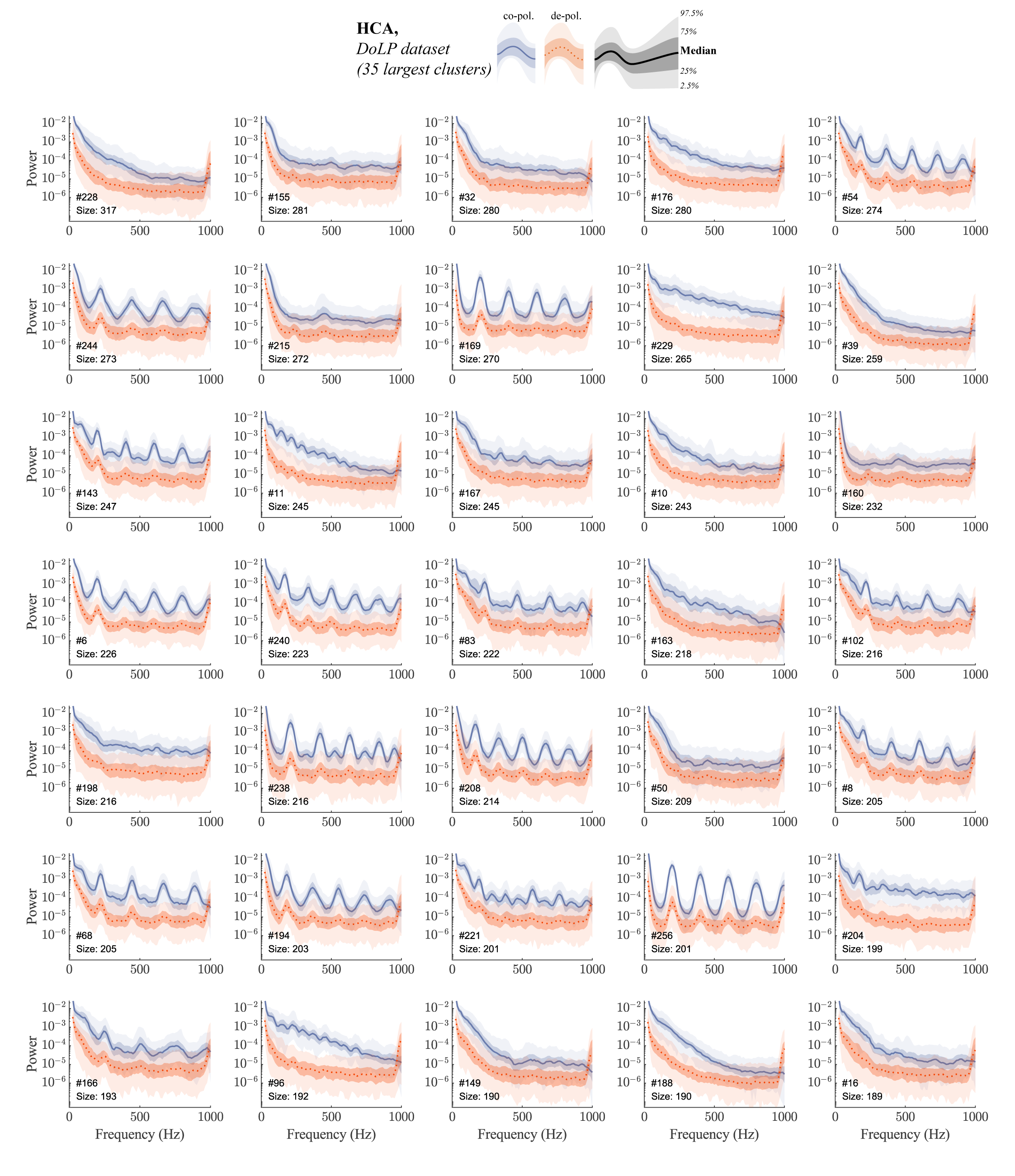

Supplement: S2 Fig — (TIF) [file pone.0312770.s002.tif]

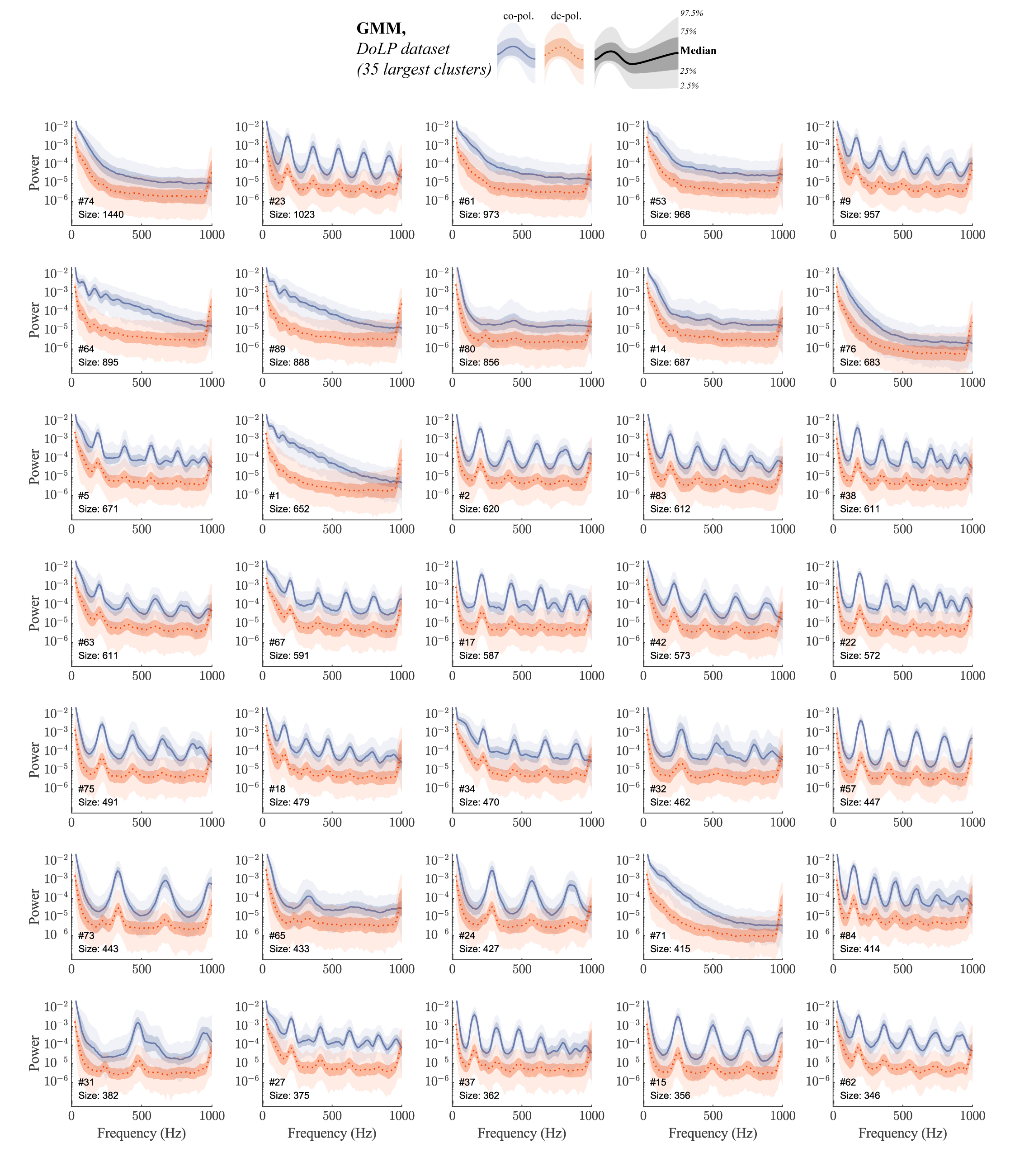

Supplement: S3 Fig — (TIF) [file pone.0312770.s003.tif]

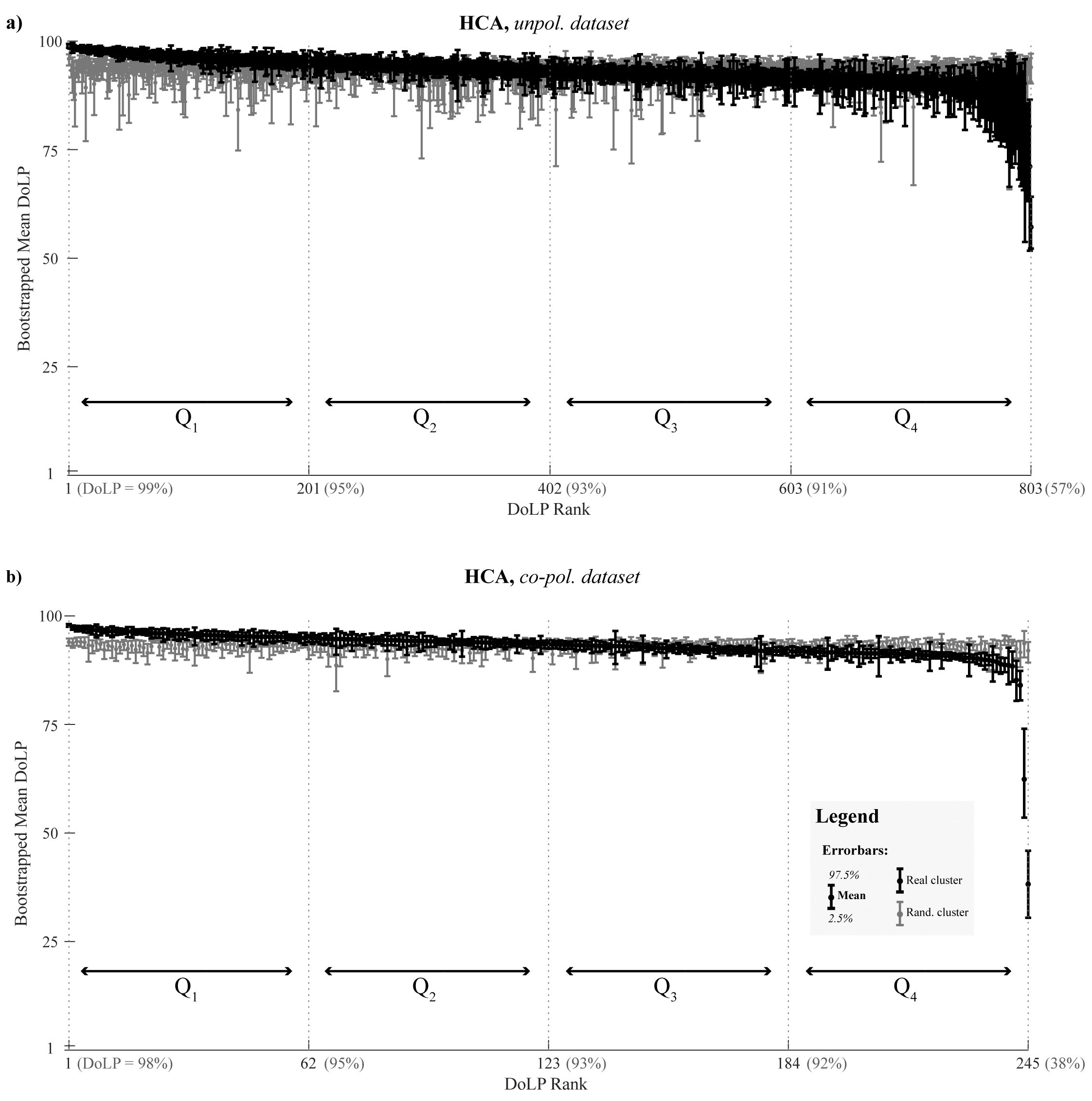

Supplement: S4 Fig — Comparison of HCA clustering results (black) with random clustering (gray). (TIF) [file pone.0312770.s004.tif]

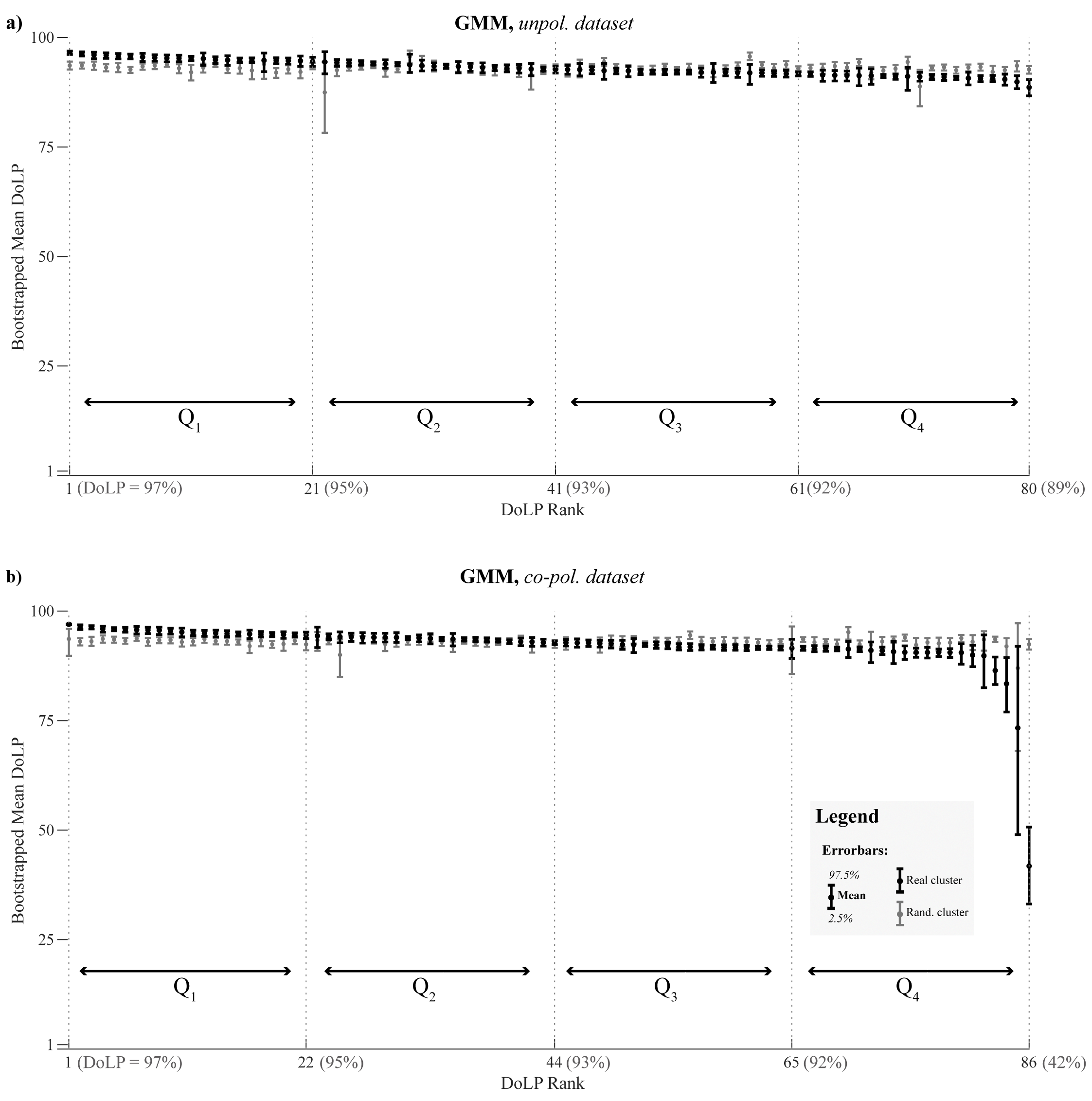

Supplement: S5 Fig — Comparison of DoLP clustering results (black) with random clustering (gray). (TIF) [file pone.0312770.s005.tif]

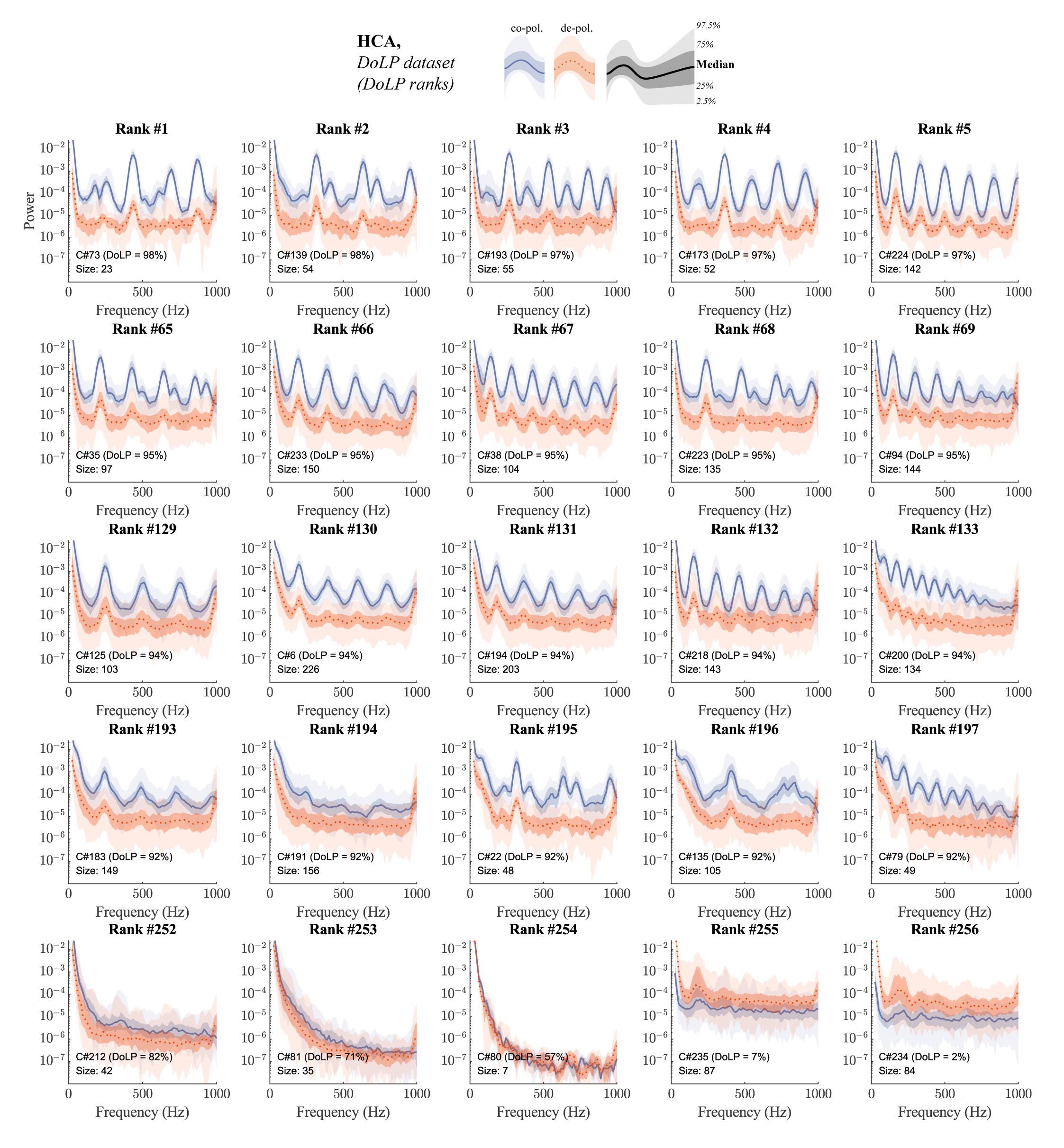

Supplement: S6 Fig — (TIF) [file pone.0312770.s006.tif]

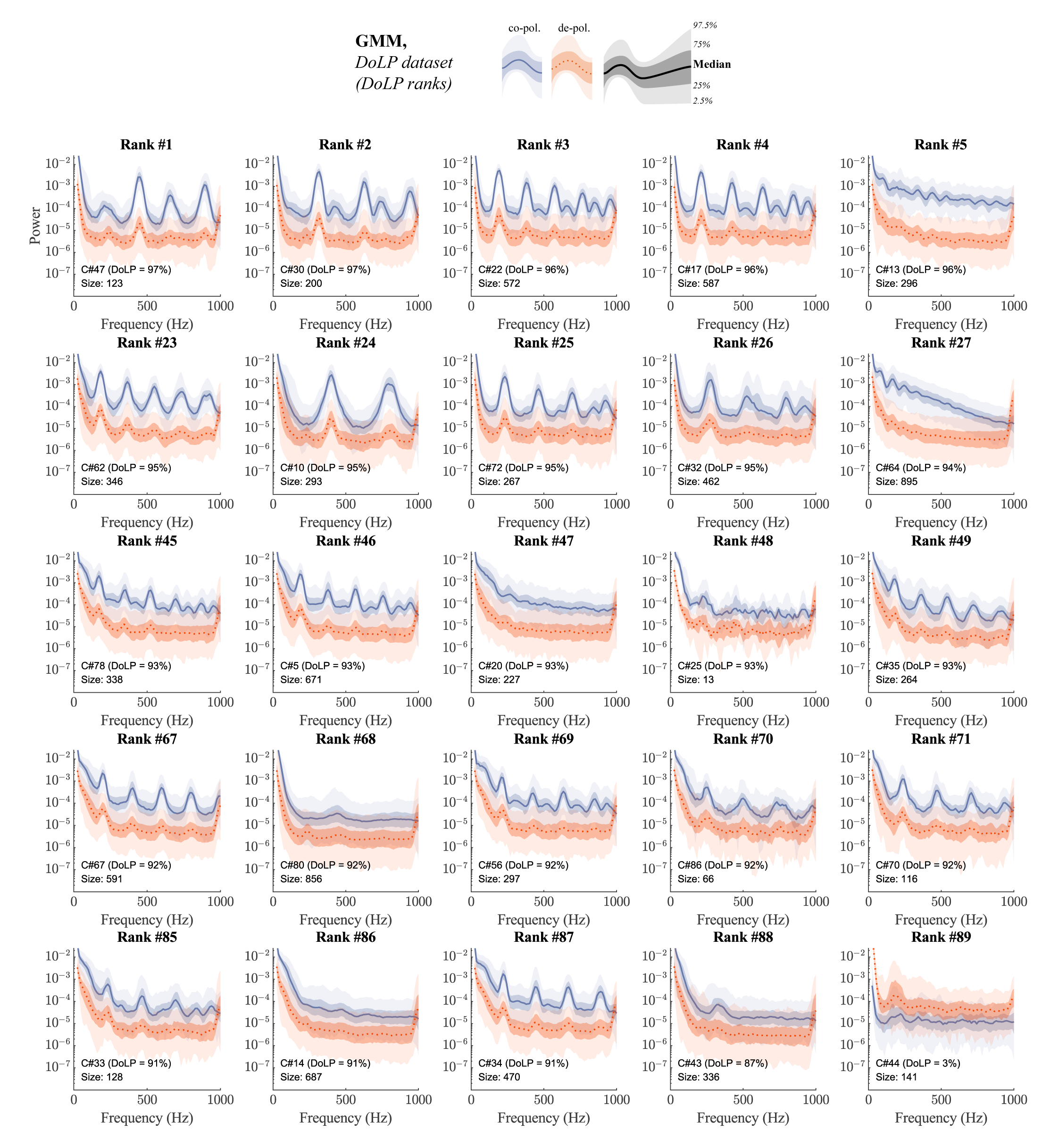

Supplement: S7 Fig — (TIF) [file pone.0312770.s007.tif]

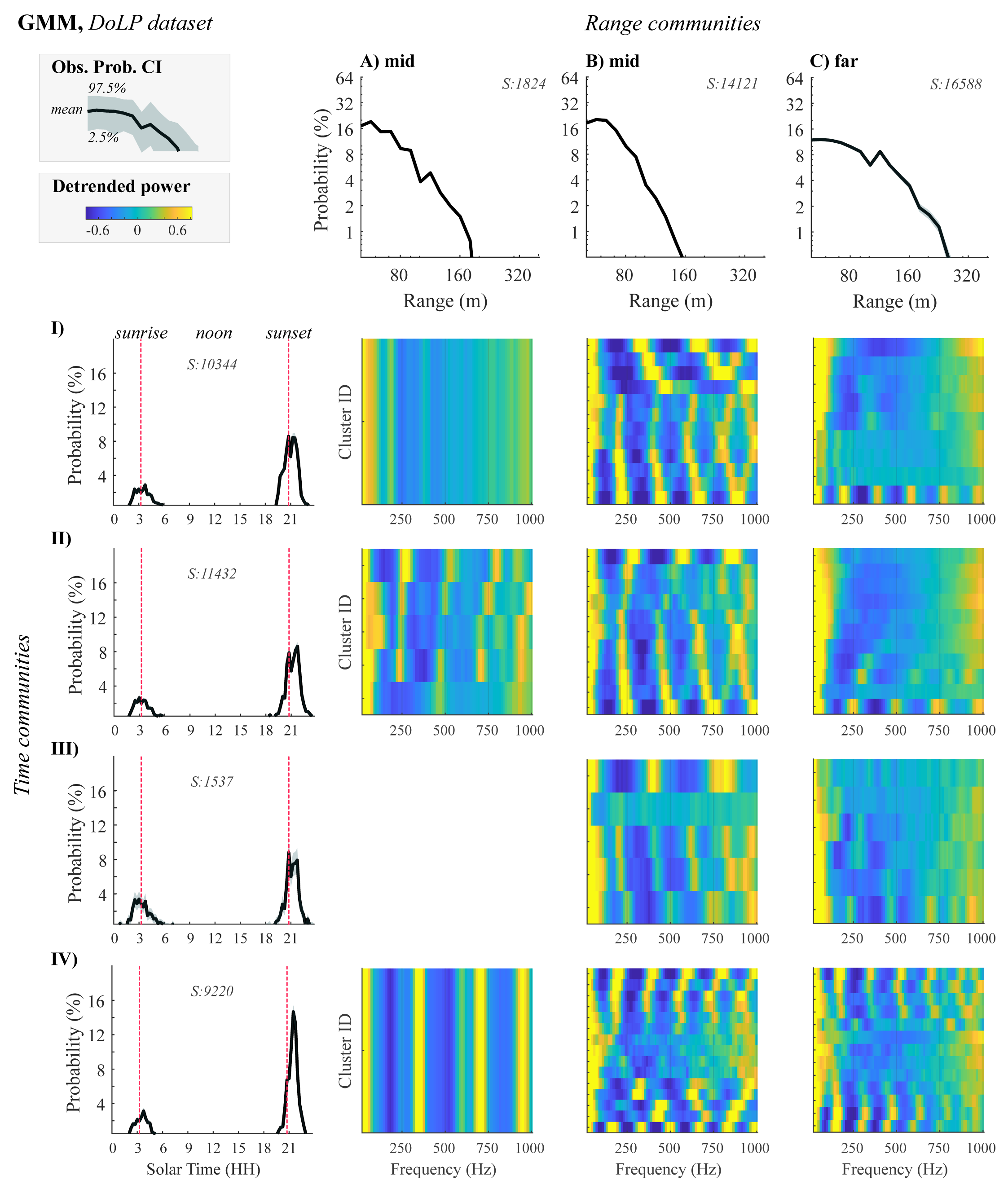

Supplement: S8 Fig — Probability distributions for range (ABC) and time (I-II-III) communities. Heatmaps at the ABC and I-II-III intersection display median power spectra for each time-range community. (TIF) [file pone.0312770.s008.tif]

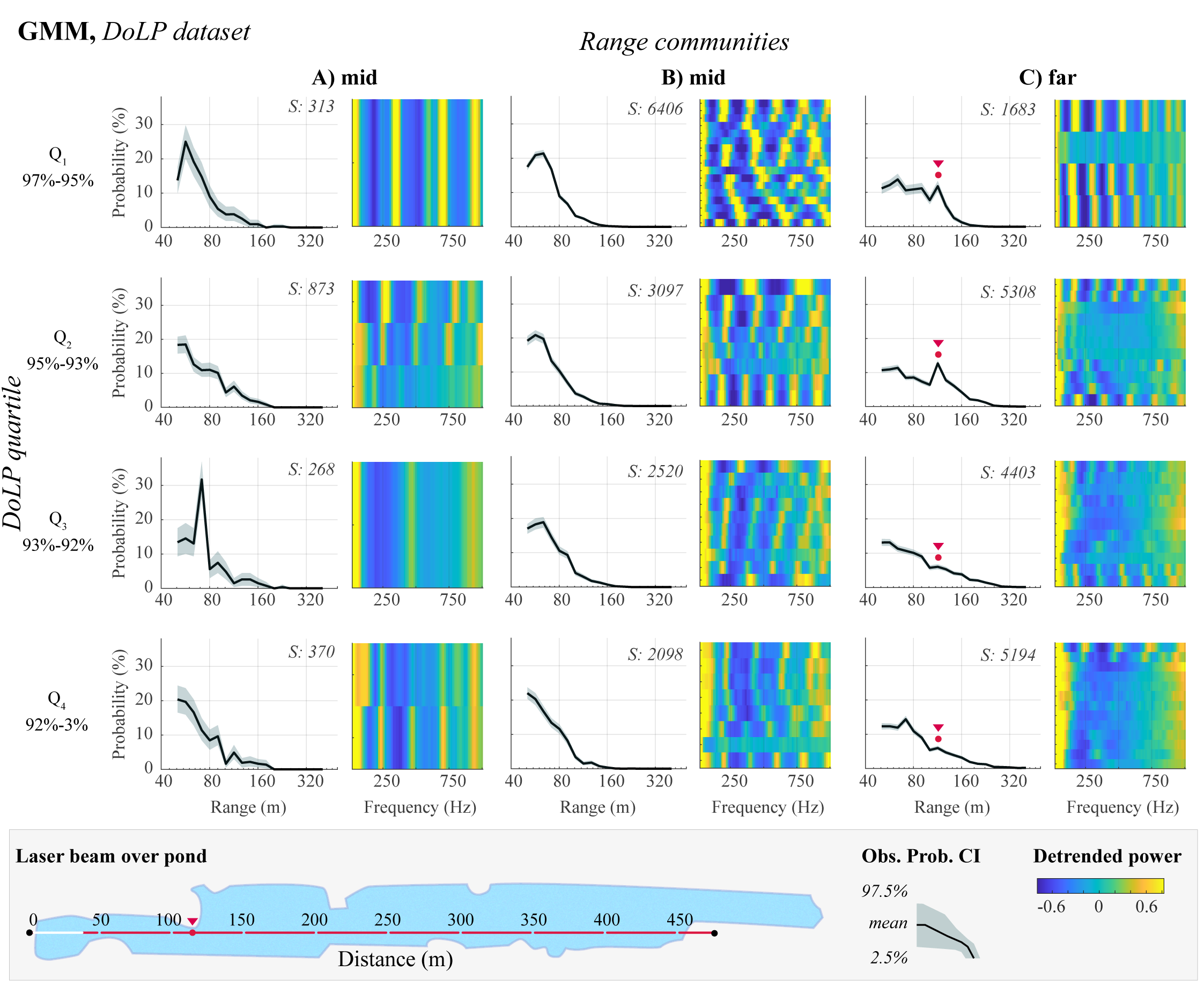

Supplement: S9 Fig — Probability distributions show the likelihood of observations within range communities (A, B, C) and DoLP quartiles (Q1-Q4), with heatmaps of corresponding power spectra. Note the probability spike in C-plots (red dot) co-occurred with the land piece left of the laser beam over the pond. (TIF) [file pone.0312770.s009.tif]
