# Supplementary material for: Insect diversity estimation in polarimetric lidar
Source: PLoS One. 2024 Nov 1;19(11):e0312770. doi: 10.1371/journal.pone.0312770 (PMC11530007; doi:10.1371/journal.pone.0312770)
Supplement: S1 Text — (DOCX) [file pone.0312770.s010.docx]

##### S1 Text. Diversity indices variability due to random UMAP/GMM initialization

We also investigated the impact of random initialization of the UMAP and GMM algorithms on clustering results. Given they are stochastic, each algorithms’ execution can yield different labeling solutions, potentially affecting the consistency of derived diversity indices.

To quantify variability of diversity indices, we perform 100 runs of UMAP embedding followed by GMM clustering. Each run, we change the random seed for both algorithms, while maintaining other parameters as described in Section **Methods: GMM**. Specifically, we keep the same value for the number of components, as was found to be optimal when using BIC score (see **S1 Table**). We summarize the results of these 100 UMAP/GMM runs in **S2 Table**, presenting the mean diversity indices in bold, along with their corresponding confidence intervals in italics (2.5^th^ and 97.5^th^ percentiles).

**S1 Table. Optimal solutions from UMAP and GMM algorithms initialized with ‘random seed’ = 42 (result reported in the main text).**

|  | Dataset | Number of components (GMM model) | Number of clusters found, *NoC, (H_0_)* | BIC  (for optimal solution) |
| --- | --- | --- | --- | --- |
| GMM | un-pol | 80 | 80 | 2.091e+05 |
|  | co-pol | 87 | 86 | 2.040e+05 |
|  | DoLP | 89 | 89 | 2.020e+05 |

**S2 Table. Variations in diversity indices resulting from 100 random initializations of UMAP and GMM.**

|  | Dataset | *H_0_, N_cl_* | *H’* | *H_1_* | *H_2_* | BIC |
| --- | --- | --- | --- | --- | --- | --- |
| GMM | un-pol | **79.34** *77...80* | **4.13** *4.05...4.19* | **62** 57.17…66.02 | **54.25** *47.85...59.22* | **2.108e+05**  *2.082e+05...2.136e+05* |
|  | co-pol | **85.44** *82...87* | **4.18** *4.10...4.24* | **65.16** *60.14…69.29* | **56.51** *50.24...61.63* | **2.059e+05** *2.034+05...2.088e+05* |
|  | DoLP | **87.34** *84...89* | **4.19** *4.11...4.27* | **66.02** *60.81...71.51* | **56.43** *51.22...62.21* | **2.050e+05** *2.023e+05...2.081e+05* |

We observed that the number of found clusters ($H_{0}$) varied by up to ±2 across all three datasets, with significantly fewer clusters found in the unpolarized dataset compared to the co-polarized and DoLP datasets. Despite these minor fluctuations in $H_{0}$, random initialization introduced variability of approximately ±5 cluster for the effective ($H_{1}$) and dominant ($H_{2}$) cluster numbers. These results show that the unpolarized and DoLP datasets differ significantly in the number of clusters ($H_{0}$), and therefore, the DoLP dataset shows a higher richness of signal. However, the variability of other indices is too high to confidently determine if these diversity estimates are significantly different between datasets.

By considering both optimal and suboptimal clustering solutions (as indicated by the BIC variability in **S2 Table**), this analysis provided insights into the stability and robustness of diversity indices in the presence of stochastic algorithms. Furthermore, it allowed us to assess whether the three datasets exhibited distinct diversity profiles, irrespective of the specific clustering solution obtained in each run.
